# Contribution of microbial activity and vegetation cover to the spatial distribution of soil respiration in mountains

**DOI:** 10.3389/fmicb.2023.1165045

**Published:** 2023-06-15

**Authors:** Sofia Sushko, Lilit Ovsepyan, Olga Gavrichkova, Ilya Yevdokimov, Alexandra Komarova, Anna Zhuravleva, Sergey Blagodatsky, Maxim Kadulin, Kristina Ivashchenko

**Affiliations:** ^1^Laboratory of Carbon Monitoring in Terrestrial Ecosystems, Institute of Physicochemical and Biological Problems in Soil Science, Pushchino, Russia; ^2^Department of Soil Physics, Physical Chemistry and Biophysics, Agrophysical Research Institute, Saint Petersburg, Russia; ^3^Center for Isotope Biogeochemistry, University of Tyumen, Tyumen, Russia; ^4^Research Institute on Terrestrial Ecosystems, National Research Council, Porano, Italy; ^5^National Biodiversity Future Center, Palermo, Italy; ^6^Laboratory of Soil Carbon and Nitrogen Cycles, Institute of Physicochemical and Biological Problems in Soil Science, Pushchino, Russia; ^7^Terrestrial Ecology Group, Institute of Zoology, University of Cologne, Cologne, Germany; ^8^Soil Science Faculty, Lomonosov Moscow State University, Moscow, Russia

**Keywords:** soil CO_2_ emission, altitudinal gradient, forest and grassland ecosystems, soil properties, plant community structure

## Abstract

The patterns of change in bioclimatic conditions determine the vegetation cover and soil properties along the altitudinal gradient. Together, these factors control the spatial variability of soil respiration (*R*_S_) in mountainous areas. The underlying mechanisms, which are poorly understood, shape the resulting surface CO_2_ flux in these ecosystems. We aimed to investigate the spatial variability of R_S_ and its drivers on the northeastern slope of the Northwest Caucasus Mountains, Russia (1,260–2,480 m a.s.l.), in mixed, fir, and deciduous forests, as well as subalpine and alpine meadows. *R*_S_ was measured simultaneously in each ecosystem at 12 randomly distributed points using the closed static chamber technique. After the measurements, topsoil samples (0–10 cm) were collected under each chamber (*n* = 60). Several soil physicochemical, microbial, and vegetation indices were assessed as potential drivers of *R*_S_. We tested two hypotheses: (i) the spatial variability of *R*_S_ is higher in forests than in grasslands; and (ii) the spatial variability of *R*_S_ in forests is mainly due to soil microbial activity, whereas in grasslands, it is mainly due to vegetation characteristics. Unexpectedly, *R*_S_ variability was lower in forests than in grasslands, ranging from 1.3–6.5 versus 3.4–12.7 μmol CO_2_ m^−1^ s^−1^, respectively. Spatial variability of *R*_S_ in forests was related to microbial functioning through chitinase activity (50% explained variance), whereas in grasslands it was related to vegetation structure, namely graminoid abundance (27% explained variance). Apparently, the chitinase dependence of *R*_S_ variability in forests may be related to soil N limitation. This was confirmed by low N content and high C:N ratio compared to grassland soils. The greater sensitivity of grassland *R*_S_ to vegetation structure may be related to the essential root C allocation for some grasses. Thus, the first hypothesis concerning the higher spatial variability of *R*_S_ in forests than in grasslands was not confirmed, whereas the second hypothesis concerning the crucial role of soil microorganisms in forests and vegetation in grasslands as drivers of *R*_S_ spatial variability was confirmed.

## 1. Introduction

Soil respiration (*R*_S_) is one of the major fluxes of the global carbon (C) cycle affecting atmospheric CO_2_ concentrations. The process of *R*_S_ potentially provides feedback to global climate change due to the large amount of C currently stored in soil organic matter (SOM), otherwise known as soil organic carbon (SOC) (Reichstein et al., 2003; Frey et al., 2013). SOC accounts for approximately 1,500 Pg of total C unequally distributed in the uppermost meter of the global soil layer, representing the largest terrestrial C pool (Kutsch et al., 2010; Schaufler et al., 2010; Mayer et al., 2020). The ratio between SOC accumulation in terrestrial ecosystems and SOC loss as CO_2_ efflux from soil determines whether an ecosystem serves as an atmospheric sink or source of C (Schlesinger and Andrews, 2000; Bolstad et al., 2004).

The processes and factors affecting *R*_S_ has become a heavily scrutinized topic in the international research community in the broad context of its efforts to mitigate long-term climate change (Bu et al., 2012; Leifeld et al., 2013). However, the extremely high temporal and spatial variability of *R*_S_ remains a challenge for the development of accurate regional and global models of the C cycle (Bond-Lamberty and Thomson, 2010). The temporal variation of the *R*_S_ process is studied worldwide and can be effectively predicted by the dynamics of soil temperature and moisture (Luo and Zhou, 2006; Xu and Shang, 2016; Hursh et al., 2017; He et al., 2023). However, much remains unclear about the spatial variation of *R*_S_ within different ecosystems, landscapes, and biomes, making it difficult to predict.

*R*_S_ composed of autotrophic (mainly root respiration) and heterotrophic components hampers the quantification of spatial drivers controlling the total CO_2_ efflux from soil (Chen et al., 2017). The contribution of each of these components is characterized by high spatial heterogeneity (Subke et al., 2006). More specifically, the spatial variation of root respiration is affected by vegetation, i.e., by species composition, abundance of herbaceous species, and the corresponding density of fine roots in the upper soil layer (Rodeghiero and Cescatti, 2008). At the same time, changes in the microbial capacity to decompose SOM (enzymatic activity, basal respiration, microbial biomass abundance, etc.) from site to site determine the spatial distribution of heterotrophic respiration (Ananyeva et al., 2020). Consequently, the drivers of spatial distribution of different components of *R*_S_ are interdependent, and it is quite difficult to determine which of these components are key.

Mountain landscapes occupy almost 25% of the global surface area (Kapos et al., 2000) and accumulate significant amounts of SOM (Canedoli et al., 2020); consequently, their contribution to the global С cycle can be considerable (Hansson et al., 2021). As for regional models describing the C cycle in mountains, the contribution of *R*_S_ accounts for up to 21% of regional emissions estimates, exceeding even that of the C-rich soil of steppes (Kudeyarov et al., 2007). Nevertheless, there is a significant lack of data on R_S_ in mountainous areas (Kudeyarov and Kurganova, 2005; Liu et al., 2014). These shortcomings are related to difficulties in logistics and equipment delivery, unfavorable climatic conditions, and measurement features (Lin et al., 2017). For instance, when measuring *R*_S_ on mountain slopes, the correction factor on steepness should be applied (Xu and Shang, 2016). Therefore, researchers usually select a flat plot to determine *R*_S_, resulting in a lack of data about mountain landscapes with steep slopes. Hence, an extension of the regional database is necessary for better understanding the spatial patterns of *R*_S_ in mountains and its drivers.

Most mountains (e.g., Himalayas, Alps, Caucasus) are covered with forests, which are replaced by grasslands at increased altitudes (Bardelli et al., 2017; Ahmad et al., 2020; Ivashchenko et al., 2021). Hence, the distribution patterns of *R*_S_ change at higher altitudes. A higher variation coefficient in forests than in grasslands has been shown due to the high heterogeneity of soil conditions caused by gaps in forest canopies (Rodeghiero and Cescatti, 2008; Katayama et al., 2009; Qi et al., 2010; Darenova et al., 2016; Shi et al., 2019). Besides, considering that the ratio of heterotrophic to autotrophic respiration in forests is mainly higher than in grasslands as reviewed by Hanson et al. (2000), it would be reasonable to assume a different contribution of microbial and vegetation properties to the spatial variations of *R*_S_ in these ecosystems. Despite a number of works having been conducted on the determination of drivers of spatial patterns of *R*_S_ in forests (Luan et al., 2012; Jiang et al., 2020), there is still a lack of clear understanding on how they are combined in different ecosystem types. Such assessments face the substantial challenge of organizing simultaneous measurements of R_S_ across various ecosystems to avoid the effect of temporal variability (Jiang et al., 2020). The spatial variability of *R*_S_ could be assayed using manipulation experiments or monitoring natural environmental gradients in the field. Manipulation experiments in controlled environments negatively affect the ability to predict system responses to changing factors and, accordingly, the capacity to effectively schedule and implement conservation actions (Pressey et al., 2007; Reside et al., 2017; Ettinger et al., 2019). Interpreting the data from monitoring experiments performed in natural environments is a more challenging issue; nonetheless, it is a promising alternative to manipulation experiments as more realistic assessments of *R*_S_ and its drivers could be achieved.

Thus, we developed an experimental design that allows for the mitigation of the effect of temporal variability via simultaneous measurements in various ecosystems along altitudinal gradient and only focused on spatial factors within forests and grasslands of the examined mountain slope. The following hypotheses were formulated:

- The spatial variability of *R*_S_ in forests is higher than in grasslands due to the high heterogeneity of environmental conditions (e.g., temperature and litter thickness) caused by canopy gaps compared to open grasslands.
- The spatial variability of *R*_S_ in forests is attributable mainly to soil microbial activity, while spatial variability of *R*_S_ in grasslands is attributable mainly to vegetation properties, taking into account different contributions of heterotrophic and autotrophic components to this process for the two ecosystem types.

## 2. Materials and methods

### 2.1. Study area and experimental design

**Figure 1:**
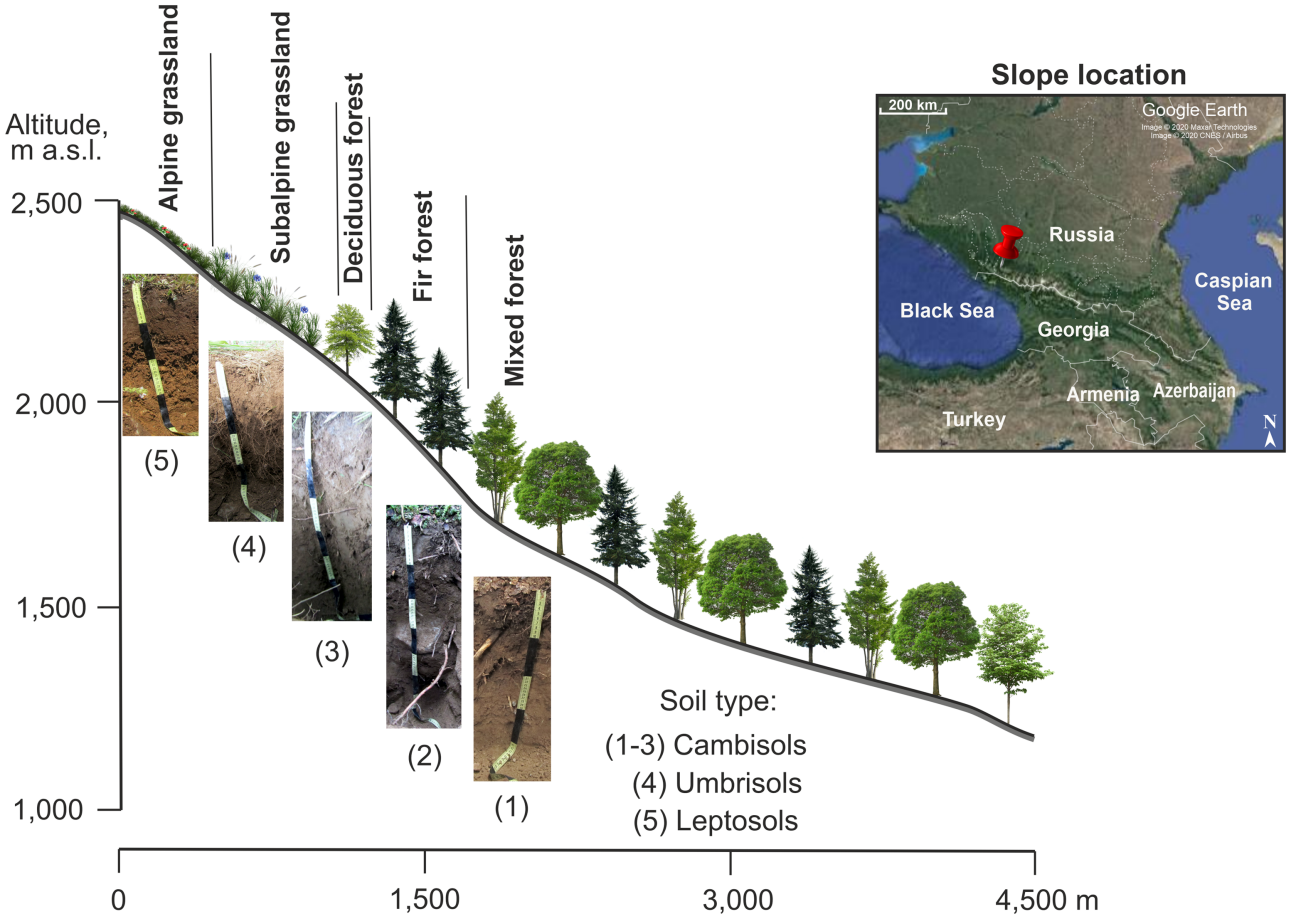
Scheme of the northeastern slope of Mt. Tkachiha (Northwest Caucasus, Karachay-Cherkess Republic, Russia).

The study was conducted in the Northwest Caucasus Mountains (43°40’N; 40°47′E) in Karachay-Cherkess Republic, South Russia. The mountain slope-to-test was the northeastern exposure and covered five vegetation belts: mixed forest at 1,200–18,00 m a.s.l., fir forest at 1,800–2,040 m a.s.l., deciduous forest at 2,040–2,290 m a.s.l., subalpine meadow at 2,290–2,300 m a.s.l., and alpine meadow at 2,300–2,500 m a.s.l. (Figure 1). The soil types were Cambisols, Umbrisols, and Leptosols formed on non-alkaline bedrock. The mean annual air temperature ranged from 3.5 to 5.9°C (our data for years 2018–2021; Table 1) and annual precipitation ranged from 800 to 1,850 mm (the nearest meteorological stations were located at 1,313 m a.s.l. and 2,006 m a.s.l.). The dominant vegetation species along the studied slope are shown in Table 1.

**Table 1:** General characteristics of studied forest and grassland sites along the northeastern slope of Mt. Tkachiha.

Site	Altitude, m a.s.l.	Slope, °	MAT, °C		Dominant vegetation
			Air	Soil	
Forests					
mixed	1,260	7	5.9	NA	*Fagus orientalis, Abies nordmanniana, Picea orientalis*
fir	1,960	20	3.4	3.9	*Abies nordmanniana*
deciduous	2,060	26	4.1	3.6	*Acer trautvetteri, Sorbus aucuparia, Betula pendula*
Grasslands					
subalpine	2,240	9	4.8	4.0	*Calamagrostis arundinacea, Festuca ovina*
alpine	2,480	6	3.5	3.2	*Carex* sp.*, Vaccinium vitis-idaea*

MAT, mean annual temperature for years 2018–2021; NA, not available.

In each vegetation belt, 12 plots of 0.5 × 0.5 m were randomly established as described by Ivashchenko et al. (2021). In total, there were 60 plots along a 1.2 km altitudinal gradient. R_S_ was measured simultaneously at all plots from 9:30 to 10:30 a.m. on August 11, 2018. This design was aimed at estimating the drivers of spatial variation of *R*_S_ without considering the causes of its temporal changes. The measurement time (day and hour) was chosen so that the primary drivers of temporal variability of *R*_S_ (i.e., plant phenology, soil temperature, and moisture) were close to representative throughout the altitudinal gradient. First, most plant species along the study slope reach their maximum phytomass and ripening stage in the first half of August. Second, on the eastern slope, there is enough light during the morning for photosynthesis, but there are still no large spatial fluctuations in soil temperature. On the measurement day, soil moisture across the slope was equally sufficient due to preceding rain events typical for the summer season at the location. Vegetation and soil physicochemical and microbial properties characterizing the sources, conditions, and mediators of CO_2_ formation in soil were considered as potential drivers of spatial variability of *R*_S_. In this case, only the upper 10-cm soil layer was taken into account, since (i) its microbial properties showed a close correlation with R_S_ for different soils and ecosystems (Sushko S. et al., 2019; Sushko S. V. et al., 2019), and (ii) it provides main portion of surface CO_2_ fluxes from moist soils (Wiaux et al., 2015).

Vegetation surveys to identify plant species and their projective cover for the herbaceous layer were carried out at each 0.5 × 0.5 m plot before *R*_S_ measurements. Simultaneously with the R_S_ measurement, soil temperature was recorded at a 10 cm depth using the Checktemp sensor (Hanna Instruments, Germany), after which a single composite sample per plot (mixing 5 cores with Ø 5 cm) was taken from the upper 0–10 cm layer, placed in a plastic bag, and transported to the lab. Fresh samples (*n* = 60) were immediately sieved through a 2 mm mesh to exclude roots, debris, stones, and homogenized them. A portion of each soil sample was used to determine its moisture by the gravimetric method (8 h, 105°C). The remained soil was used for microbial and chemical analysis. Subsamples for microbial analysis were stored at 4°C for up to 2 weeks after sampling.

### 2.2. Soil respiration

Soil respiration (*R*_S_), i.e., CO_2_ efflux from the soil surface, was measured by the closed static chamber technique. For this, non-transparent PVC chambers (Ø 15.5 cm, volume 1.8 L) were inserted into the soil to a depth of 2–3 cm (above-ground grass was preliminarily cut) perpendicular to the slope surface. Three air samples (20 ml) were taken from each chamber through a rubber stopper with a gas-tight syringe and analyzed with an infrared gas analyzer (SBA-5, PP system, USA). The first zero-time sample was collected immediately after installing the chamber, while the other two were collected at an interval of 3–5 min. The day before, preliminary measurements were performed with gas sampling at nine time points (0, 1, 2, 3, 4, 5, 10, 15, 20 min) to determine the period of initial linear increasing of CO_2_ concentration inside the closed chamber at each site. It was found that 5 min is a representative time for all ecosystems during which the gas increases linearly (mean *R*^2^ = 0.98 ± 0.03). Additionally, we recorded atmospheric pressure along the altitudinal gradient simultaneously with R_S_ measurements using a meteorological barometer. The R_S_ rate (μmol CO_2_ m^−2^ s^−1^) was calculated according to the following equation:


(1) $$R_{S}=\frac{\mathrm{VP}}{\mathrm{RST}}\times \frac{\partial C}{\partial t},$$

where *V* is chamber volume (m^3^), *P* is air chamber pressure (Pa), *R* is gas constant (8.314 m^3^ Pa K^−1^ mol^−1^), *S* is soil surface area (m^2^), *T* is chamber air temperature (K), and *∂С/∂t* is change of CO_2_ concentration inside the chamber over time (μmol mol^−1^ s^−1^). The final R_S_ rate was corrected for the surface slope angle in degrees (*θ*): *R*_S_/cos *θ* (Xu and Shang, 2016).

### 2.3. Soil and vegetation analysis

Soil total C and N contents were determined by the dry combustion method using a CHNS analyzer (Leco Corp., USA). Dissolved organic carbon (DOC) and dissolved total nitrogen (DTN) were determined in 0.05 M K_2_SO_4_ extracts (5 g soil: 20 mL solution) using a Shimadzu TOC-VCPN analyzer (Shimadzu Corp., Japan) (Makarov et al., 2015). The pH was measured in a soil:water suspension (1:2.5 ratio) with a conductivity Sartorius Basic Meter (Germany). Microbial biomass carbon (MBC) was measured by the substrate-induced respiration method (Anderson and Domsch, 1978; International Organization for Standardization, 1997). Basal respiration (BR) was measured as the rate of soil CO_2_ release using gas chromatography (KrystaLLyuks-4,000 M; Meta-Chrom, Russia) (International Organization for Standardization, 2002). The MBC and BR values were determined under optimum hydrothermal conditions for the microorganisms: 22°C and 65% water holding capacity. Three hydrolytic C-and N-acquiring enzymes (β-d-glucosidase, chitinase, and leucine aminopeptidase) were measured using fluorogenic substrates (Marx et al., 2005; International Organization for Standardization/Technical Specification, 2019) as detailed by Ivashchenko et al. (2021). The soil samples for MBC, BR, and enzyme activities were pre-incubated at 22°C for 72 h (Loeppmann et al., 2016).

We chose characteristics of the herbaceous layer as a potential driving factor along the altitudinal gradient because: (i) it is a uniform ecological plant group for forest and grassland sites; (ii) despite a small contribution to overall forest biomass, this plant strata significantly affect N, P, K, and Mg cycles and mediate C dynamics at the ecosystem level (Gilliam, 2007). For each of the plots studied, the number of species (richness) and total projective coverage for graminoids and forbs were estimated (Ivashchenko et al., 2021). These functional plant groups were the most abundant and presented throughout the altitudinal gradient. In addition, the Shannon-Wiener plant diversity index (H_plant_) for the herbaceous layer was calculated using the following equation:


(2) $$H_{\mathrm{plant}}=-\sum p_{i}\ln p_{i}$$

where p*i* is the ratio of *i* species projective cover to total projective cover of all species per plot.

### 2.4. Statistical analysis

The spatial variability of the studied properties within forests and grasslands was quantified by the coefficient of variation (CV, %), calculated as the ratio of the standard deviation to the mean. Additionally, boxplots combined with dot plots were used to show the variability of *R*_S_ across the studied sites. The significance of differences in variables between two or more independent groups was tested by Welch’s *t*-test or one-factor analysis of variance (ANOVA), respectively. Principal component analysis (PCA) was used to show variations and relationships between the studied environmental variables, as well as to illustrate the difference between forest and grassland sites. To reveal possible drivers of *R*_S_ spatial variability within forests and grasslands, we used forward stepwise regression and path analysis. First, significant factors within each group of variables (soil physicochemical, microbial, and vegetation) were identified by forward stepwise regression using permutation tests. Afterward, the causal relation between the identified predictors was examined using path analysis. The pre-analysis and preparation of data included (1) checking the normality of distribution with a histogram plot and Shapiro–Wilk’s test, (2) log-transformation of some variables to achieve a normal distribution (i.e., C, N, C:N, DOC, BR, enzyme activity), and (3) centering and scaling to unit variance. All analyses and visualizations were performed in the R software system (version 4.1.2, RStudio Team, 2023) using the following packages: “ggplot2” for boxplots (Wickham, 2016), “FactoMineR” (Lê et al., 2008) and “factoextra” (Kassambara and Mundt, 2020) for PCA, “packfor” for forward stepwise regression (Dray et al., 2016), and “lavaan” for path analysis (Rosseel, 2012).

## 3. Results

### 3.1. Environmental characteristics in forests and grasslands

As expected, soil environments essentially differed between mountain forests and grasslands (Table 2). Grasslands located at higher altitudes than forests accumulated more organic matter (in the C, N, DOC, DTN forms) in soils characterized by a lower C:N ratio. Under both types of vegetation, the soils were strongly acidic (pH 5.0–5.2). Soil temperature in forests varied noticeably higher than in grasslands; its average values were 14.1, 11.3, 11.6, 13.7, and 13.0°C for mixed, fir, deciduous forests, subalpine and alpine grasslands, respectively. Conversely, water content was more homogenous in forests than in grasslands, averaging 39.3, 38.8, 39.4, 45.3 and 61.2% for the above listed ecosystems, respectively. Among the physicochemical properties studied in forest and grassland soils, the DTN content showed the highest spatial variability (CV 55–63%).

**Table 2:** Topsoil (0–10 cm) and vegetation (herbaceous layer) characteristics of forest and grassland sites along the studied mountain slope.

Variables	Forests (*n* = 35)		Grasslands (*n* = 24)	
	Mean ± SE	CV, %	Mean ± SE	CV, %
C, g kg^−1^	73 ± 4	30	162 ± 11***	32
N, g kg^−1^	5.1 ± 0.2	28	13.1 ± 0.8***	31
DOC, μg g^−1^	127 ± 6	29	206 ± 8***	19
DTN, μg g^−1^	59 ± 6	63	95 ± 11**	55
C:N	14.3 ± 0.3	14	12.2 ± 0.2***	8
pH	5.2 ± 0.1	10	5.0 ± 0.1	7
Temperature, °C	12.3 ± 0.2	10	13.4 ± 0.1***	5
WC, %	39 ± 1	22	53 ± 2**	20
MBC, μg C g^−1^	1,883 ± 97	30	4,475 ± 247***	27
BR, μg C g^−1^ h^−1^	2.57 ± 0.18	42	3.62 ± 0.27**	36
*β*-Glucosidase†	1.28 ± 0.24	112	2.95 ± 0.39***	64
Chitinase	2.96 ± 0.68	137	7.18 ± 1.53*	104
LAP^1^	6.71 ± 1.11	98	0.27 ± 0.05***	83
Coverage, %	18 ± 2	73	94 ± 2***	9
Graminoids, %	2 ± 1	164	44 ± 5***	56
Forbs, %	11 ± 2	110	34 ± 3***	47
Richness	5	54	7 **	27
*H*_plant_	0.94 ± 0.08	50	1.32 ± 0.08**	31

Values present means ± standard error (SE) with a coefficient of variation (CV); *P ≤ 0.05; **0.01; ***0.001 for Welch’s *t*-test. C, total carbon; N, total nitrogen; DOC, dissolved organic carbon; DTN, dissolved total nitrogen; WC, water content; MBC, microbial biomass carbon; BR, basal respiration; LAP, leucine aminopeptidase; Richness, number of species; H_plant_, Shannon–Wiener diversity index. ^†^Unit for enzyme activity is μmol MUF g^−1^ h^−1^ (*β*-glucosidase, chitinase) or μmol AMC g^−1^ h^−1^ (LAP).

Differences in contents of total and dissolved forms of C and N between the forest and grassland soils led to significant differences in their microbial properties. Basically, indices of microbial activity in grasslands exceeded those in forests, except for leucine aminopeptidase (LAP) activity. Among the soil microbial properties, the activity of enzymes had the highest spatial variation within both forests and grasslands (CV 64–137%).

The forest understory (18% projective coverage) was formed mostly by forbs (11%). By contrast, the projective coverage (>90%) of grasslands almost equally consisted of graminoids and forbs. As a result, the number of species and the homogeneity of their distribution within a plot (H_plant_) were higher in grasslands than in forests. In general, the spatial variation of all of the vegetation properties studied was considerably higher in forests than in grasslands (CV 50–164% vs. 9–56%).

**Figure 2:**
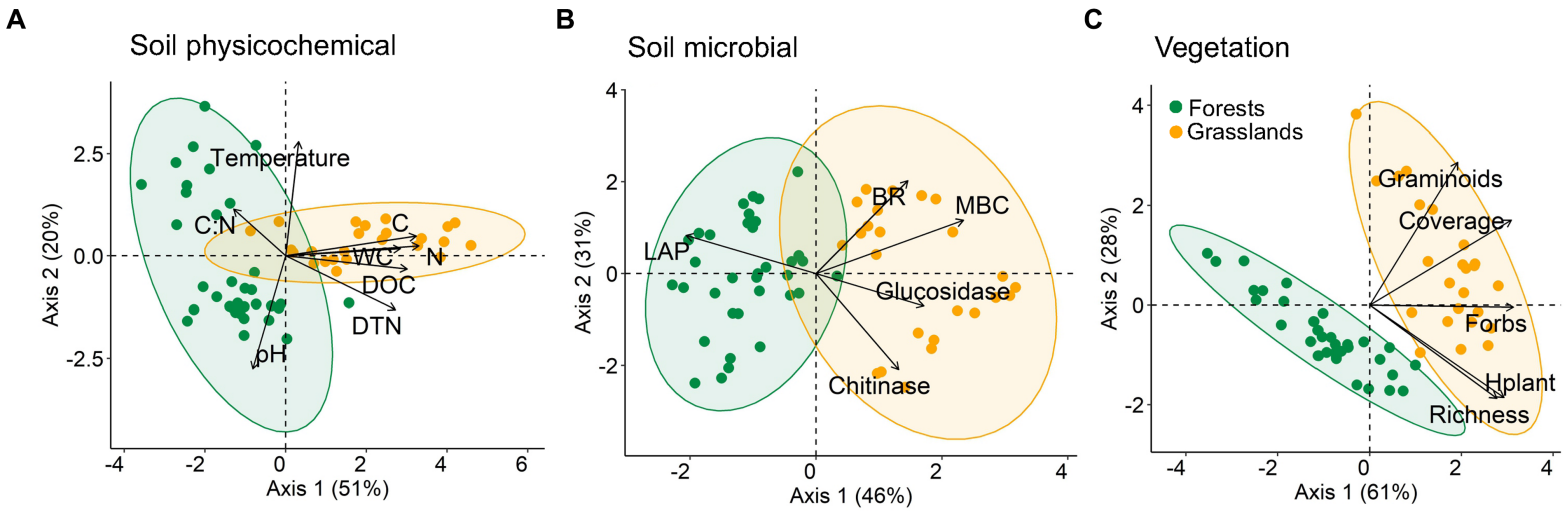
Principal component analysis (PCA) ordination triplot for soil physicochemical **(A)**, soil microbial **(B)**, and vegetation **(C)** characteristics of mountain forest and grassland sites. See variable abbreviations in Table 2.

The PCA analysis for all measured characteristics showed a clear grouping of studied sites by vegetation type, i.e., forests and grasslands (Figures 2A,B). Among the soil physicochemical parameters, the organic matter (in the C, N, DOC, DTN forms) and water contents mostly differed between forest and grassland soils (allocation of points along axis 1; *r^2^* = 0.64–0.92) (Figure 2A). It is worth noting that the temperature and pH value of forest soils were characterized by extremely high variance (allocation of points along axis 2; *r*^2^ = 0.64–0.65), while for grassland soils they were more homogeneous. Equally high variation for soil microbial parameters was found within both forests and grasslands (Figure 2B). At the same time, soils under these vegetation types were separately grouped along axis 1, mainly according to MBC content and LAP activity (*r*^2^ = 0.76 and 0.59, respectively). In terms of herbaceous layer features, forest and grassland sites were clearly separated from each other mainly along axis 1, which is associated with variations in almost all of the studied characteristics (total and forbs projective coverage, number of species, *H*_plant_; *r*^2^ = 0.60–0.76) (Figure 2C).

### 3.2. Spatial variation of soil respiration and its possible drivers

**Figure 3:**
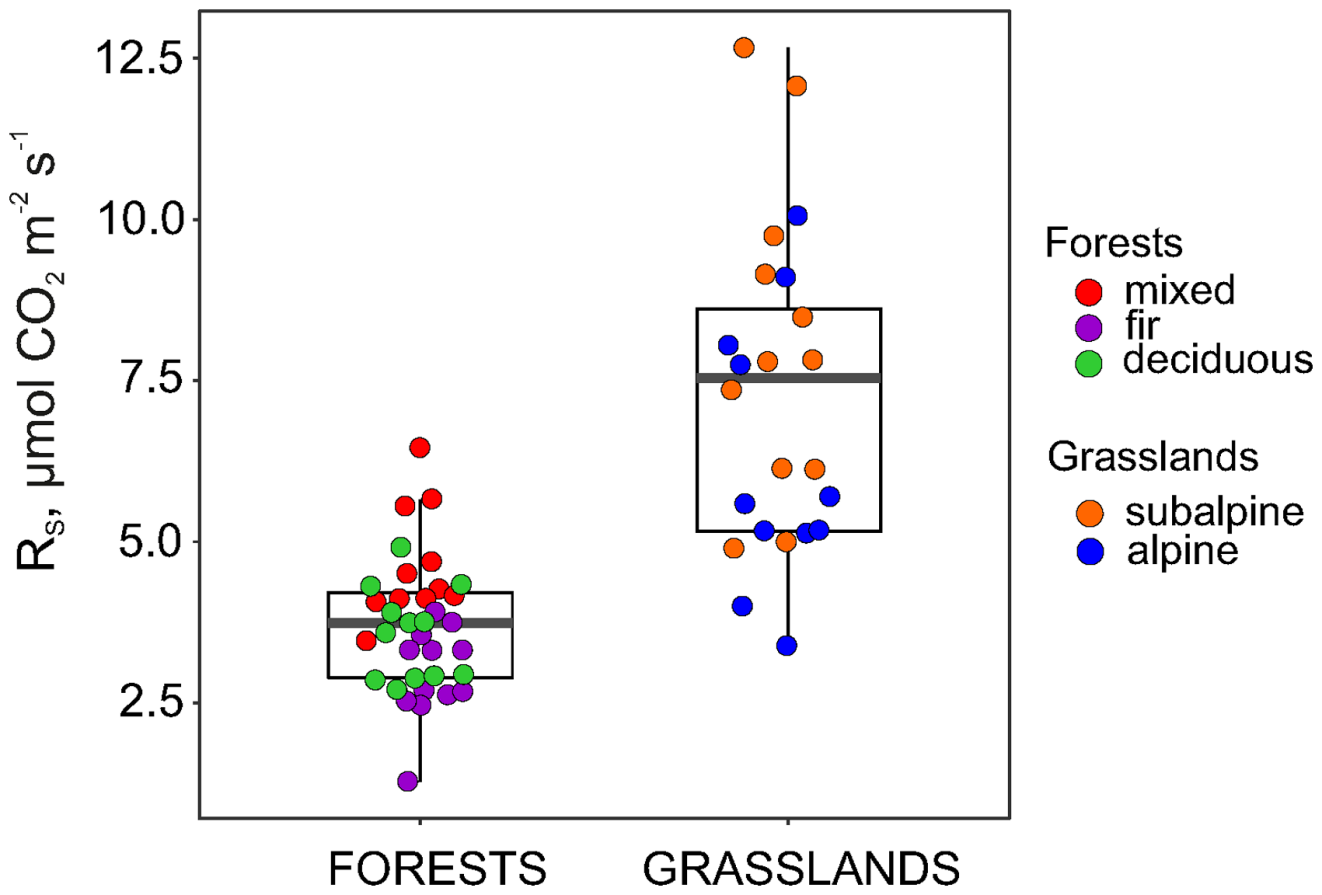
Soil respiration (*R*_S_) for mountain forest and grassland sites along altitudinal gradient.

The *R*_S_ rate in the forests was, on average, half of that in the grasslands, reaching 3.7 and 7.3 μmol CO_2_ m^−1^ s^−1^, respectively (Figure 3). Within forests, *R*_S_ increased significantly in the following order: fir < deciduous < mixed forests with average values of 3.0, 3.6 and 4.6 μmol CO_2_ m^−1^ s^−1^, respectively (*p* < 0.001 for one-way ANOVA). At the same time, *R*_S_ did not differ between subalpine and alpine grasslands with average values of 8.1 and 6.4 μmol CO_2_ m^−1^ s^−1^, respectively (*p* = 0.09 for Welch’s *t*-test). In general, the spatial variation of R_S_ in forests was lower than that in grasslands (CV 28 and 33%, respectively).

Difference in *R*_S_ rate between forests and grasslands was associated with their divergence in soil (C, N, DOC, temperature, MBC and enzyme activities; *R*^2^ = 0.23–0.45, *p* ≤ 0.001) and vegetation properties (coverage and graminoid abundance; *R*^2^ = 0.49 and 0.55, *p* ≤ 0.001) (Supplementary Figure S1). Notably, the relationship with vegetation were stronger than with soil conditions, indicating its primary role in determining *R*_S_ rate across ecosystem types.

Stepwise regression analysis showed that *R*_S_ spatial variation in forests was best predicted by soil temperature, chitinase activity, and species richness of the herbaceous layer (explained variation 29–50%) (Table 3). The best predictors for grasslands included chitinase activity and graminoid abundance (explained variation 19 and 27%, respectively).

**Table 3:** Results of stepwise regression for *R*s dependence on soil and vegetation characteristics in mountain forest and grassland sites.

Variables	Forests (*n* = 35)		Grasslands (*n* = 24)	
	*R* ^2^	*F-*value	*R* ^2^	*F-*value
C	0.01	0.81	0.02	0.47
N	0.02	1.31	0.01	0.34
DOC	0.00	0.14	0.00	0.01
DTN	0.03	2.31	0.08	1.99
C:N	0.02	1.37	0.11	2.62
pH	**0.07**	5.03*	0.00	0.03
Temperature	**0.45**	26.54***	0.00	0.07
Water content	0.06	4.58	0.04	0.93
MBC	0.03	1.94	0.05	1.44
BR	0.01	0.74	0.07	1.89
β-Glucosidase	0.01	0.73	0.05	1.41
Chitinase	**0.50**	32.72***	**0.19**	5.09*
LAP	0.05	3.37	0.04	1.30
Coverage	0.00	0.09	0.04	1.10
Graminoids	0.01	0.23	**0.27**	8.19**
Forbs	0.02	0.71	0.01	0.29
Richness	**0.29**	12.79*	0.01	0.57
Hplant	0.02	0.71	0.02	0.79

For each variable group, the analysis was performed separately. See variable abbreviations in Table 2. Bold indicates significant values at *p ≤ 0.05; **0.01; ***0.001.

**Figure 4:**
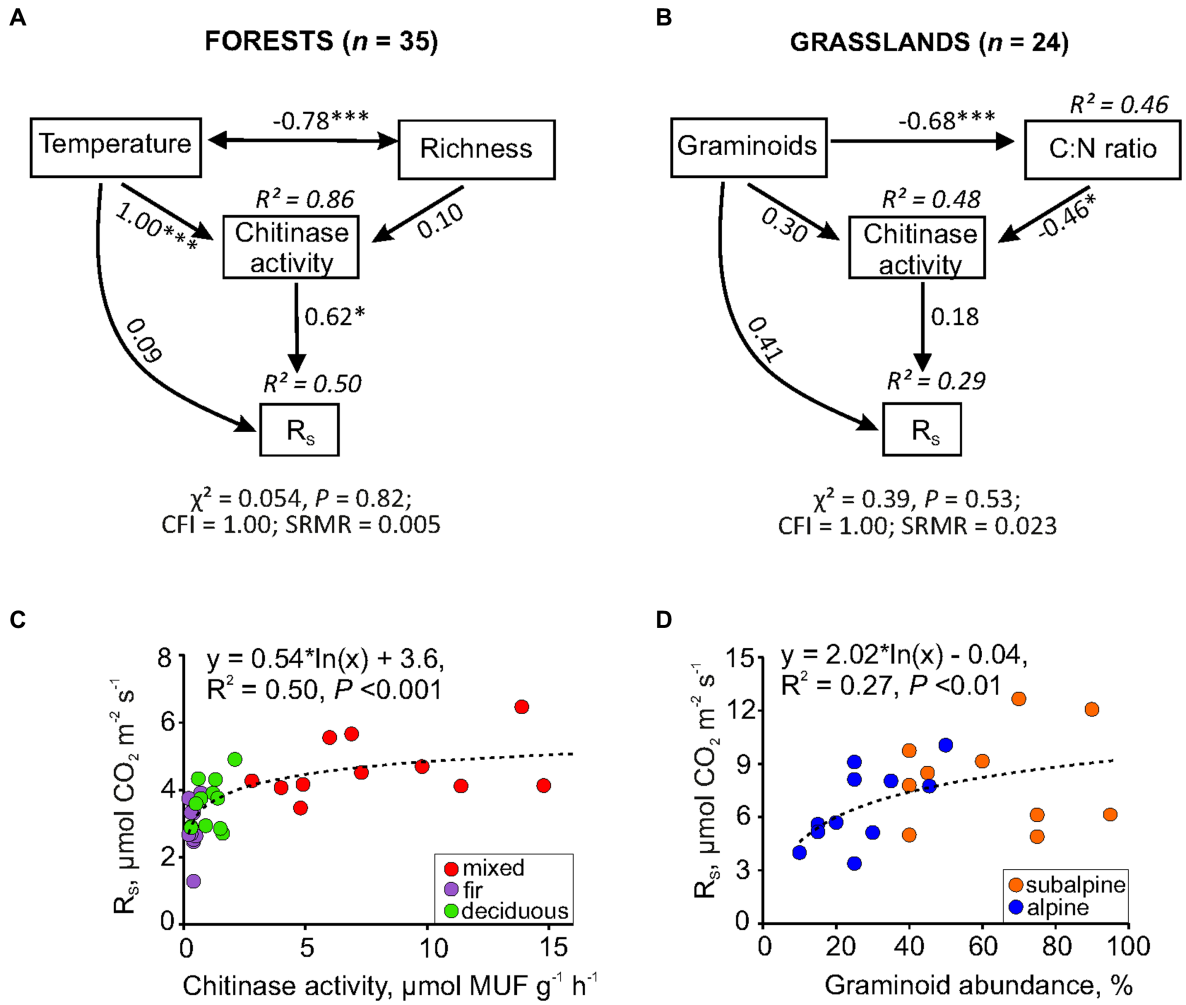
Path models and simple regression revealed the effects of soil (temperature, chitinase activity, C:N ratio) and vegetation factors (species richness, graminoid abundance) on *R*_S_ spatial variability in mountain forests **(A,C)** and grasslands **(B,D)**. In the path model, numbers within double-headed arrows are correlation coefficients between variables, numbers within one-way arrows are standardized path coefficients indicating the size effect of the causal relationship among variables (*p ≤ 0.05; **0.01; ***0.001); CFI, comparative fit index; SRMR, standardized root mean square residual.

Subsequent path analysis was used to explore the direct or indirect effects of the identified predictors on R_S_ spatial variation. For forests, soil temperature was included in the path model both as (i) a direct factor, due to changing physical components of *R*_S_, e.g., gas pressure and its diffusive transport throughout pore space, and (ii) an indirect factor, acting through the regulation of chitinase activity. The plant species richness was considered only as an indirect factor since it determines the quality of the organic substrate entering the soil (root exudates, above-and below-ground litter), which, in turn, can affect chitinase activity. In general, the path model explained 50% of *R*_S_ variation across forest sites (Figure 4A). In turn, soil temperature affected *R*_S_ indirectly through the regulation of chitinase activity. Plant species richness did not affect chitinase, showing only a strong negative correlation with temperature.

For grasslands, chitinase activity, graminoid abundance, and, additionally, C:N ratio as their linking factor (indicated on the base correlation among all variables; see Supplementary Table S1) were included in the path model. As a result, the proposed model explained 29% of the *R*_S_ variation in grasslands (Figure 4B). As expected, graminoids affected chitinase activity via changes in the soil C:N ratio. However, the significance of direct or indirect effects of graminoids and chitinase activity on *R*_S_ was not confirmed by the model despite its overall satisfactory goodness-of-fit. Nevertheless, the largest standardized path coefficient (0.41) was found for the direct effect of graminoids on *R*_S_. This fact can likely explain the more pronounced effect of vegetation features on grassland *R*_S_ variability than soil characteristics associated with the microbial decomposition of SOM (e.g., chitinase activity).

Summarizing the results of all of the analyses performed, one can conclude that the spatial changes in *R*_S_ within mountain forests were mainly driven by soil chitinase activity alone, while spatial changes in *R*_S_ in grasslands were associated with graminoid abundance. These relationships have been additionally demonstrated using a simple regression (Figures 4C,D).

## 4. Discussion

### 4.1. Spatial variability of soil respiration in mountain forests and meadows

Our results showed a higher *R*_S_ rate in grasslands than in forests, which is consistent with the earlier reported difference between these ecosystem types (Raich and Tufekciogul, 2000; Kao and Chang, 2009). Meanwhile, intra-ecosystem *R*_S_ variability was also higher in grasslands than in forests (Figure 3), which contradicts both the data reported for other mountain regions (Rodeghiero and Cescatti, 2008; Darenova et al., 2016) and our first hypothesis. On the one hand, this discrepancy can be attributed to the different time scales of measurements and, accordingly, different sources of *R*_S_ variation. Specifically, our data show only the spatial variation in *R*_S_ at individual time points without including its fluctuations during a season or year (i.e., combined spatio-temporal variability), unlike that in the above-mentioned studies. On the other hand, high altitude grasslands adopt accelerated photorespiration to complete growth and development within a short growing season (Streb and Cornic, 2012). Alpine species reach maximum photosynthetic capacity at a wide range of light intensity (i.e., 530–3,000 μmol photons m^−2^ s^−1^) with a higher interspecific variation than in lowland plants (Korner and Diemer, 1987). This phenomenon, along with a species-specific time lag between photosynthesis and root-derived CO_2_ efflux from soil (Kuzyakov and Gavrichkova, 2010), could have caused high spatial variability in *R*_S_ within grasslands in our study.

### 4.2. Drivers of spatial variability in soil respiration in mountain forests and meadows

In our study, the drivers of spatial variability in *R*_S_ were fundamentally different between forests and grasslands. In forests, both biotic and abiotic factors influenced the spatial variability of *R*_S_. A primary biotic driver was soil chitinase activity (Figure 4). The chitinase is one of the key enzymes of the C-and N-cycling, catalyzing degradation of chitin and peptidoglycan to carbohydrates and inorganic N available for soil microorganisms and plants (Andersson et al., 2004). With an increase in the availability of substrates for maintenance and growth of microorganisms and plants, their enzyme production would diminish, and vice versa (Allison et al., 2010). The relationship between chitinase activity and *R*_S_ in forests could be explained by soil N deficiency, which is confirmed by significantly low contents of total and dissolved N forms, as well as by a high C:N ratio (Table 2). At the same time, the N-related enzyme LAP did not affect *R*_S_, unlike chitinase (Table 3). A reasonable explanation for this finding is that LAP catalyzes the hydrolysis of amino acids (Sinsabaugh et al., 2009), which is a less important source of N than amino sugars (i.e., chitin) contained in microbial necromass (Buckeridge et al., 2022). The main abiotic driver of the spatial variability in forest *R*_S_ was soil temperature, which acted indirectly through the regulation of chitinase (Figure 4). The lower temperatures stimulate microbial necromass accumulation that would decompose primarily with increase in temperature, in particular by the chitinase (Bai et al., 2016; Wang et al., 2021). Moreover, a dominant portion of microbial necromass in forest topsoil is represented by fungi, which is an essential source of chitin and other amino sugars (Ni et al., 2020; Wang et al., 2021). Accordingly, in our study, a strong relationship between temperature and chitinase was found at colder soil conditions in forests compared to grasslands (Table 2). Thus, chitinase activity and its dependence on biotic and abiotic factors is critically important considering the inter-relations between C-and N-cycling in forest soils.

In grasslands, spatial variability in *R*_S_ was related to vegetation structure, especially graminoid abundance, rather than chitinase activity (Table 3 and Figure 4). Overall, weaker relationships between potential drivers and *R*_S_ in grasslands unlike in forests can be attributed to the difference in the heterogeneity of the above-and below-ground vegetation structure, biomass, and C allocation. Below-ground C allocation of photosynthates in grasslands is high, especially for some graminoids (Liu et al., 2021), which can eventually cause a considerable fraction of autotrophic (root-derived) respiration in *R*_S_ (Hanson et al., 2000). Therefore, the relationship between *R*_S_ and graminoid abundance was the most pronounced in studied grasslands. An important role of the vegetation structure and functioning in spatial heterogeneity of *R*_S_ in grasslands was also confirmed by previous studies (e.g., Bahn et al., 2008; Fóti et al., 2016). At the same time, abiotic factors would be crucial for *R*_S_ changes at unfavorable temperature and water conditions, e.g., in cold and drought periods. Therefore, we can expect an enormous variation in the importance of a number of spatial drivers of *R*_S_ depending on season, as earlier reported by Shi and colleagues (Shi et al., 2020) for grassland ecosystems.

### 4.3. Prospects to study and forecast spatial variability of soil respiration

Monitoring *R*_S_ is a complicated task due to its high variability in space and time (Rodeghiero and Cescatti, 2008). The spatial variation coefficient of *R*_S_ can also be higher than that of *R*_S_ temporal variation (Rodeghiero and Cescatti, 2008; Jiang et al., 2020). Therefore, the optimization of *R*_S_ measurements to decrease uncertainties caused by spatial factors is an urgent matter. A sample size for *R*_S_ measurements could be optimized using multiple probability simulation (Rodeghiero and Cescatti, 2008). However, for such a model, comprehensive information on *R*_S_ spatial variability and its site-specific drivers is needed. Previous studies of *R*_S_ spatial variability were combined with examinations of its temporal dynamics (Martin and Bolstad, 2005; Kosugi et al., 2007; Luan et al., 2012; Jiang et al., 2020). Under such conditions, spatial and temporal variability are overlapped since the measurements for different plots by closed chamber methods take several hours to even several days (Jiang et al., 2020). Consequently, high temporal variability in *R*_S_ contributes to uncertainty in its spatial variability. The most accurate measurements are provided by long-term systems for multiplexed soil CO_2_ flux measurements, which are able to evaluate both spatial and temporal flux variations without overlapping across a large footprint (LI-COR, 2023). However, this measurement system is expensive and difficult to install on steep slopes. In our study, we sought to minimize *R*_S_ temporal variability in mountain ecosystems, and thus focused on its spatial variability, which makes our research relatively novel for such kinds of field experiments. Simultaneous air sampling from closed chambers in five ecosystems took approximately 1 h and allowed for the analysis of CO_2_ concentrations on the same day despite the time spent for climbing and descent. However, extrapolating our results in time should be performed with care because seasonal variability in *R*_S_ was not considered in the current set-up. Nevertheless, splitting the drivers for spatial patterns of *R*_S_ between forests and grasslands constitutes a useful integration for C-cycle models (Jackson et al., 2017). Moreover, projecting our results to the consequences of climate change allowed us to assay not only C turnover changes but also drivers of its spatial variability at treeline shifts in mountains; therefore, further research in this direction appears to be a promising endeavor (Chen et al., 2022).

## 5. Conclusion

This study provides a comprehensive analysis of vegetation and soil factors along the altitudinal gradients to identify potential drivers of *R*_S_ spatial variability. The results have shown principal differences in *R*_S_ spatial patterns and their drivers between forest and grassland ecosystems. A higher spatial variability of this process was found in grasslands than in forests, which contradicts our first hypothesis. Soil microbial activity (i.e., chitinase) contributed greatly to spatial variability of *R*_S_ in forests, while vegetation (graminoid abundance) was more considerable factors in grasslands, which fully confirmed our second hypothesis. These findings highlighted how CO_2_ fluxes in mountain forest and grassland ecosystems are regulated by a number of biotic and abiotic factors, emphasizing the most probable mechanisms of mutual regulations of C-and N-cycles.

## Data availability statement

The raw data supporting the conclusions of this article will be made available by the authors, without undue reservation.

## Author contributions

SS and KI: conception of the study. SS, KI, LO, AK, MK, and AZ: field measurements and laboratory analysis. SS, KI, LO, OG, SB, and IY: data interpretation, result discussion, and writing original draft. All authors contributed to the article and approved the submitted version.

## Funding

Analytical processing and modeling were supported by state assignment No. 122111000095–8. Data survey was supported by Russian Science Foundation No. 22–74-10124.

## Conflict of interest

The authors declare that the research was conducted in the absence of any commercial or financial relationships that could be construed as a potential conflict of interest.

## Publisher’s note

All claims expressed in this article are solely those of the authors and do not necessarily represent those of their affiliated organizations, or those of the publisher, the editors and the reviewers. Any product that may be evaluated in this article, or claim that may be made by its manufacturer, is not guaranteed or endorsed by the publisher.
