# Supplementary material for: Contribution of microbial activity and vegetation cover to the spatial distribution of soil respiration in mountains
Source: Front Microbiol. 2023 Jun 15;14:1165045. doi: 10.3389/fmicb.2023.1165045 (PMC10307969; doi:10.3389/fmicb.2023.1165045)
Supplement: Supplementary file 1 [file Data_Sheet_1.docx]

Supplementary Material

Contribution Of Microbial Activity And Vegetation Cover To The Spatial Distribution Of Soil Respiration In Mountains

Sofia Sushko, Lilit Ovsepyan, Olga Gavrichkova, Ilya Yevdokimov, Alexandra Komarova, Anna Zhuravleva, Sergey Blagodatsky, and Kristina Ivashchenko*

*** Correspondence:** Corresponding Author: ivashchenko.kv@gmail.com

Table S1. Pearson’s correlation coefficient (***P ≤0.01****, n* = 35) between studied variables (Var) for mountain forests.

| Var | C | N | DOC | DTN | C:N | pH | T | WC | MBC | BR | Glu | Chit | LAP | Cover | Gram | Forbs | Rich | H_plant_ |
| --- | --- | --- | --- | --- | --- | --- | --- | --- | --- | --- | --- | --- | --- | --- | --- | --- | --- | --- |
| C | 1.00 |  |  |  |  |  |  |  |  |  |  |  |  |  |  |  |  |  |
| N | ***0.90*** | 1.00 |  |  |  |  |  |  |  |  |  |  |  |  |  |  |  |  |
| DOC | ***0.47*** | ***0.51*** | 1.00 |  |  |  |  |  |  |  |  |  |  |  |  |  |  |  |
| DTN | ***0.45*** | ***0.68*** | ***0.66*** | 1.00 |  |  |  |  |  |  |  |  |  |  |  |  |  |  |
| C:N | 0.38 | 0.00 | -0.04 | ***-0.47*** | 1.00 |  |  |  |  |  |  |  |  |  |  |  |  |  |
| pH | 0.11 | 0.24 | ***0.51*** | ***0.69*** | -0.35 | 1.00 |  |  |  |  |  |  |  |  |  |  |  |  |
| T | -0.28 | ***-0.49*** | -0.40 | ***-0.81*** | ***0.44*** | ***-0.62*** | 1.00 |  |  |  |  |  |  |  |  |  |  |  |
| WC | 0.21 | 0.23 | 0.19 | 0.08 | 0.06 | 0.14 | 0.02 | 1.00 |  |  |  |  |  |  |  |  |  |  |
| MBC | ***0.54*** | ***0.59*** | ***0.51*** | ***0.67*** | -0.04 | ***0.49*** | ***-0.66*** | 0.10 | 1.00 |  |  |  |  |  |  |  |  |  |
| BR | ***0.59*** | ***0.49*** | ***0.53*** | 0.32 | 0.39 | 0.29 | -0.34 | 0.25 | ***0.71*** | 1.00 |  |  |  |  |  |  |  |  |
| β-Glu | 0.18 | 0.28 | 0.25 | ***0.46*** | -0.37 | 0.40 | -0.16 | 0.02 | 0.25 | -0.14 | 1.00 |  |  |  |  |  |  |  |
| Chit | -0.13 | -0.34 | -0.21 | ***-0.63*** | ***0.46*** | ***-0.50*** | ***0.93*** | 0.12 | ***-0.52*** | -0.24 | 0.00 | 1.00 |  |  |  |  |  |  |
| LAP | 0.32 | ***0.54*** | ***0.53*** | ***0.88*** | ***-0.54*** | ***0.72*** | ***-0.70*** | -0.03 | ***0.54*** | 0.12 | ***0.73*** | ***-0.50*** | 1.00 |  |  |  |  |  |
| Cover | 0.01 | 0.15 | 0.08 | ***0.51*** | -0.34 | 0.40 | ***-0.48*** | -0.19 | 0.33 | -0.05 | 0.15 | ***-0.48*** | ***0.45*** | 1.00 |  |  |  |  |
| Gram | -0.03 | -0.07 | -0.21 | -0.23 | 0.17 | -0.35 | 0.25 | -0.08 | -0.18 | -0.22 | -0.19 | 0.15 | -0.26 | 0.27 | 1.00 |  |  |  |
| Forbs | 0.14 | 0.34 | 0.15 | ***0.64*** | ***-0.45*** | ***0.52*** | ***-0.74*** | -0.11 | ***0.52*** | 0.16 | 0.18 | ***-0.66*** | ***0.61*** | ***0.74*** | -0.07 | 1.00 |  |  |
| Rich | 0.11 | 0.39 | 0.36 | ***0.82*** | ***-0.62*** | ***0.61*** | ***-0.80*** | -0.17 | ***0.51*** | 0.02 | ***0.44*** | ***-0.71*** | ***0.84*** | ***0.65*** | -0.07 | ***0.73*** | 1.00 |  |
| H_plant_ | -0.11 | 0.18 | 0.25 | ***0.61*** | ***-0.62*** | ***0.52*** | ***-0.68*** | -0.16 | 0.40 | 0.03 | 0.16 | ***-0.68*** | ***0.58*** | ***0.58*** | 0.04 | ***0.64*** | ***0.88*** | 1.00 |

Table S2. Pearson’s correlation coefficient (***P ≤0.01****, n* = 24) between studied variables (Var) for mountain grasslands.

| Var | C | N | DOC | DTN | C:N | pH | T | WC | MBC | BR | Glu | Chit | LAP | Cover | Gram | Forbs | Rich | H_plant_ |
| --- | --- | --- | --- | --- | --- | --- | --- | --- | --- | --- | --- | --- | --- | --- | --- | --- | --- | --- |
| C | 1.00 |  |  |  |  |  |  |  |  |  |  |  |  |  |  |  |  |  |
| N | ***0.97*** | 1.00 |  |  |  |  |  |  |  |  |  |  |  |  |  |  |  |  |
| DOC | ***0.50*** | ***0.51*** | 1.00 |  |  |  |  |  |  |  |  |  |  |  |  |  |  |  |
| DTN | ***0.82*** | ***0.78*** | ***0.66*** | 1.00 |  |  |  |  |  |  |  |  |  |  |  |  |  |  |
| C:N | ***0.62*** | 0.45 | 0.27 | ***0.62*** | 1.00 |  |  |  |  |  |  |  |  |  |  |  |  |  |
| pH | ***-0.83*** | ***-0.76*** | -0.46 | ***-0.87*** | ***-0.64*** | 1.00 |  |  |  |  |  |  |  |  |  |  |  |  |
| T | ***-0.54*** | -0.47 | -0.35 | -0.44 | ***-0.56*** | ***0.52*** | 1.00 |  |  |  |  |  |  |  |  |  |  |  |
| WC | ***0.76*** | ***0.74*** | ***0.54*** | ***0.76*** | ***0.60*** | ***-0.63*** | ***-0.55*** | 1.00 |  |  |  |  |  |  |  |  |  |  |
| MBC | 0.41 | 0.45 | ***0.50*** | 0.36 | 0.22 | -0.10 | -0.13 | ***0.54*** | 1.00 |  |  |  |  |  |  |  |  |  |
| BR | ***0.70*** | ***0.66*** | ***0.62*** | ***0.64*** | ***0.53*** | -0.45 | -0.44 | ***0.73*** | ***0.76*** | 1.00 |  |  |  |  |  |  |  |  |
| β-Glu | ***-0.65*** | ***-0.55*** | -0.36 | ***-0.55*** | ***-0.61*** | ***0.69*** | ***0.72*** | -0.46 | 0.02 | -0.43 | 1.00 |  |  |  |  |  |  |  |
| Chit | ***-0.80*** | ***-0.73*** | -0.40 | ***-0.75*** | ***-0.66*** | ***0.85*** | ***0.55*** | ***-0.59*** | -0.08 | -0.47 | ***0.79*** | 1.00 |  |  |  |  |  |  |
| LAP | ***0.78*** | ***0.73*** | 0.25 | ***0.76*** | ***0.57*** | ***-0.75*** | -0.28 | ***0.57*** | 0.10 | 0.49 | -0.44 | ***-0.65*** | 1.00 |  |  |  |  |  |
| Cover | -0.24 | -0.19 | -0.08 | -0.35 | -0.34 | 0.28 | 0.10 | -0.20 | 0.01 | -0.28 | 0.29 | 0.43 | ***-0.50*** | 1.00 |  |  |  |  |
| Gram | ***-0.57*** | -0.47 | -0.12 | ***-0.50*** | ***-0.66*** | **0.61** | 0.45 | -0.49 | 0.00 | -0.36 | 0.40 | ***0.57*** | ***-0.67*** | 0.46 | 1.00 |  |  |  |
| Forbs | 0.12 | 0.09 | -0.01 | 0.17 | 0.22 | -0.20 | 0.01 | 0.13 | -0.06 | -0.06 | 0.07 | -0.06 | 0.13 | 0.15 | ***-0.53*** | 1.00 |  |  |
| Rich | ***0.63*** | ***0.57*** | 0.28 | ***0.56*** | ***0.54*** | -0.49 | -0.10 | 0.41 | 0.35 | ***0.59*** | -0.37 | ***-0.56*** | ***0.63*** | ***-0.50*** | ***-0.66*** | 0.29 | 1.00 |  |
| H_plant_ | ***0.64*** | ***0.58*** | 0.32 | ***0.57*** | ***0.53*** | -***0.53*** | -0.21 | ***0.50*** | 0.30 | ***0.51*** | -0.26 | -0.44 | ***0.61*** | -0.25 | ***-0.83*** | ***0.62*** | ***0.84*** | 1.00 |


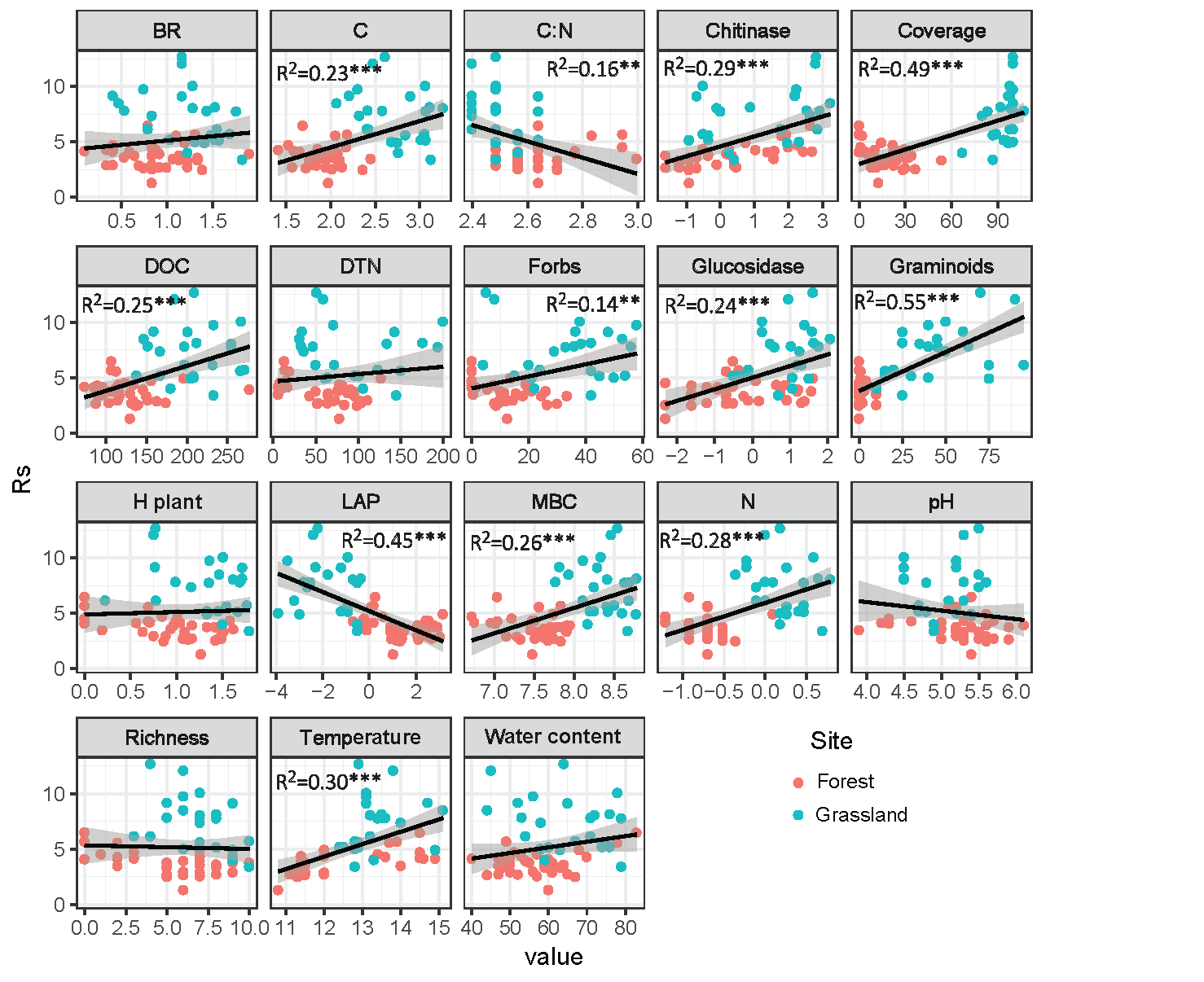


Figure S1. Linear regression between soil respiration (R_S_) and studied soil and vegetation variables along the altitudinal gradient (*n* = 59; ***P* ≤0.01; ***0.001)
